# Quantifying synergistic interactions of ternary additives for microstructural control in ultrathin Li-ion battery copper foils

**DOI:** 10.1039/d5ra06657c

**Published:** 2025-11-18

**Authors:** JinZe Zhang, Jialin Li, Liping Wang, Zhongbo Bai, Eryong Liu, Hui Cai, Jingli Zhang, Qinhao Yang

**Affiliations:** a School of Energy Power and Mechanical Engineering, North China Electric Power University Beijing 102206 China; b School of Materials Science and Engineering, Xi'an University of Science and Technology Xi'an Shaanxi 710054 China liueryong@xust.edu.cn +86-29-85587373 +86-29-85587373; c Lingbao Wason Copper Foil Co., Ltd. Lingbao Henan 472500 China

## Abstract

The advancement of lithium-ion batteries toward higher energy density and safety necessitates the development of ultrathin copper foil current collectors (such as 6 µm or thinner) with enhanced mechanical properties. However, reducing the foil thickness invariably compromises its mechanical integrity, posing safety risks. This study investigates a low-cost ternary additive system comprising 3,3′-dithiodipropanesulfonic acid disodium salt (SPS), collagen, and hydroxyethyl cellulose (HEC) for the direct current (DC) electrodeposition of 6 µm copper foils and the effects of individual and composite additives on microstructural evolution (morphology, grain orientation, and roughness) and mechanical performance (tensile strength and elongation). A quantitative correlation between the additives and the microstructure–mechanical properties of the copper foil is established. The results reveal complementary mechanisms, where SPS promotes deposit densification and grain morphology transition from conical to hill-like; collagen facilitates ultra-grain refinement; and HEC dominates surface flattening and mechanical enhancement. Through synergistic optimization, exceptional properties are achieved, where compared to the additive-free foils, the elongation increases by 229.0% (to 4.58%), the tensile strength increases by 188.2% (to 661.4 MPa), and the surface roughness decreases by 73.8% (*R*_z_ to 0.84 µm). This work not only elucidates the microscopic synergy of SPS/collagen/HEC but also proposes a scalable, cost-effective strategy for the industrial production of high-performance ultrathin copper foils.

## Abstract 2

The advancement of lithium-ion batteries toward higher energy density and safety necessitates the development of ultrathin copper foil current collectors (such as 6 µm or thinner) with enhanced mechanical properties.

## Graphical abstract

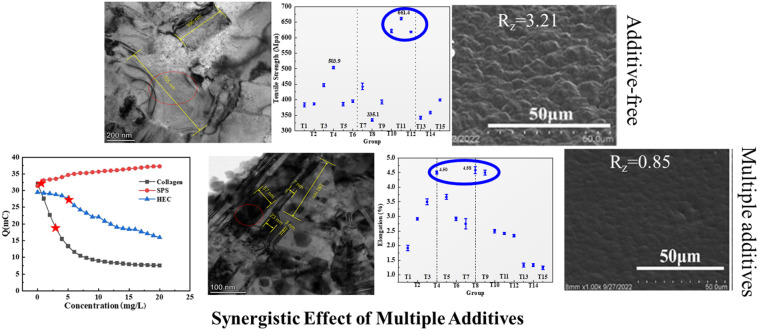

## 1. Introduction

The rapid advancement of lithium-ion batteries toward higher energy density and safety has intensified the demand for ultrathin anode current collectors. Copper foil, as the primary conductive substrate, plays a critical role in battery performance.^1–4^ Although reducing the foil thickness to 6 µm is a key strategy for enhancing the energy density, it invariably compromises the mechanical integrity due to the thickness-induced degradation of tensile strength and ductility, posing significant safety concerns.^5–8^ Consequently, the development of ultra-thin foils with high toughness has become a paramount challenge driving innovation in copper foil technology.

Traditional copper electrodeposition additives, which are classified as accelerators (*e.g.*, SPS), suppressors (*e.g.*, HEC), and levelers, function by modulating the nucleation kinetics and cathode surface adsorption.^9–19^ While binary systems (*e.g.*, SPS/HEC or SPS/protein) improve the mechanical properties of thicker foils (>8 µm), research on ternary additives for sub-6 µm copper foils remains scarce. For example, T. Nagayama *et al.* investigated the addition of three additives, bis-(3-sulfopropyl)-disulfide disodium salt (SPS), animal low molecular protein (PBF), and hydroxyethyl cellulose (HEC), and varied the amount of SPS, showing that the addition of SPS effectively improved the tensile strength and hardness of electrolytic copper foil.^20^ The research conducted by Wang *et al.* demonstrated that introducing a Tu–PAM additive in the electrolyte resulted in a notable reduction in the particle size and enhanced surface flatness of the copper foil compared to using the additive alone.^21^ Sun *et al.* demonstrated that the co-incorporation of hydroxyethyl cellulose and bis-(3-sulfopropyl)-disulfide expanded the scope of effective concentrations of sodium saccharin and enhanced the durability of the copper foil characteristics.^22^ Nevertheless, research on the quantitative understanding of the synergistic mechanisms in ternary systems that simultaneously enhance the strength, ductility, and surface quality of ultrathin foils is still lacking.^23–26^

Thus, to address this gap, this study investigates a cost-effective ternary additive system with SPS, collagen, and HEC for DC-electrodeposited 6 µm copper foils. This study focuses on quantitatively correlating the effects of individual and composite additives with microstructural evolution (morphology, grain orientation, and roughness) and macroscopic mechanical properties (tensile strength and elongation). The ultimate goal is to establish a synergistic optimization strategy that overcomes the trade-off constraints in ultrathin battery copper foils.

## 2. Experimental materials and methods

### 2.1 Electrode preparation

In this experiment, an oxygen-free copper plate (dimensions: 180 mm × 120 mm) served as the anode, which was polished with SiC sandpaper from P400 to P2000 grit to eliminate surface oxides. The plate was then cleaned *via* ultrasonic treatment in distilled water for 10 min, immersed in 10% H_2_SO_4_ for 30 s to remove residual contaminants, rinsed with distilled water, and dried under nitrogen flow until no visible water droplets remained. Industrial TA2-grade pure titanium (dimensions: 170 mm × 60 mm) was employed as the cathode, which was polished from P400 to P2000 grit, and subjected to an identical polishing and cleaning protocol.

### 2.2 Electrolyte formulation & electrodeposition process

The base electrolyte was composed of 468.17 g L^−1^ CuSO_4_·5H_2_O, 110 g L^−1^ H_2_SO_4_, and 16.5 ppm HCl. The SPS (0.7–10 ppm), collagen (5–50 ppm) and HEC (7–50 ppm) additives were introduced separately into the electrolyte. Copper foils (target thickness: 6 µm) were deposited in a thermostatically controlled Teflon-lined electrolytic cell at 55 °C ± 0.5 °C, a temperature selected to optimize Cu^2+^ ion diffusion, enhance the electrolyte conductivity, and promote the additive adsorption–desorption kinetics, thereby ensuring the formation of a uniform microstructure under high-speed deposition conditions. Electrodeposition was carried out at a constant current density of 40.0 ± 0.5 A dm^−2^, with the deposition time calibrated to achieve a thickness of 6 µm. Sixteen composite additive formulations (T0–T15) were designed based on single-additive screening results, with the specific concentration combinations of SPS, collagen, and HEC detailed in Table 1. After deposition, the copper foils were carefully peeled from the titanium cathode for subsequent microstructure characterization and mechanical property testing.

**Table 1:** Concentration of composite additives in the electrolyte

Experimental group	SPS (ppm)	Collagen (ppm)	HEC (ppm)
T0	0	0	0
T1	0.7	0.7	0.7
T2	0.7	1	1
T3	0.7	3	3
T4	0.7	3	5
T5	0.7	5	7
T6	0.7	7	10
T7	1	3	5
T8	1	5	7
T9	1	7	10
T10	1	5	10
T11	1	7	30
T12	1	10	50
T13	3	5	10
T14	3	7	30
T15	3	10	50

### 2.3 Characterization techniques

A Metrohm CVS, Switzerland was selected to explore the influence of the copper plating solution system and additives on the copper deposition amount. The surface morphology of the copper foil surfaces was imaged using field-emission scanning electron microscopy (FESEM, JSM-7900F). Arithmetic mean roughness (*R*_z_) was quantified using a contact profilometer (HXTB-ZJNJ-023) according to ISO 4287, with six measurements per sample averaged for statistical reliability. Mechanical properties (tensile strength and elongation) were evaluated on an INSTRON 3343 universal testing machine. Samples measuring 12.7 mm × 152 mm × 6 µm were cut with a precision die cutter, with a gauge length of 50 mm and a strain rate of 2 mm min^−1^. Three regions per sample were analyzed to ensure representativeness.

Crystallographic texture was tested using a Shimadzu XRD-7000 diffractometer with Cu-Kα radiation (*λ* = 1.5406 Å). Scans (2*θ* = 20–80°) were performed at 4° min^−1^ to calculate the texture coefficients (*T*_c(*hkl*)_) *via*eqn (1). TEM analysis was conducted on a Talos F200X microscope at 200 kV. The foils were thinned to electron transparency by Ar^+^ ion milling (Leica EM RES102) at 5 keV.1
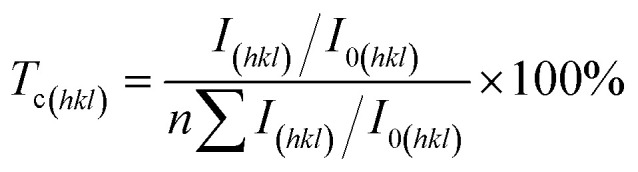
where *I*_(*hkl*)_ = measured intensity and *I*_0(*hkl*)_ = standard intensity (JCPDS 04-0836).

## 3. Results and discussion

### 3.1 Microstructure and evolution of copper foils with single additives

#### 3.1.1 Electrochemical behavior of the plating solution

**Fig. 1:**
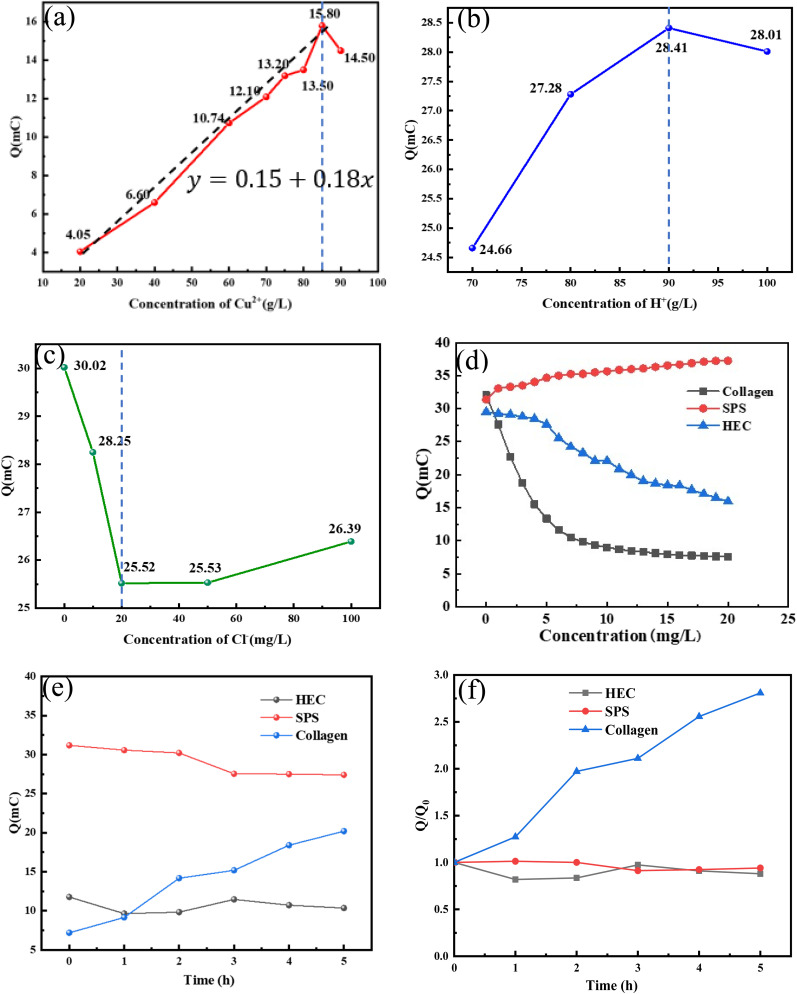
*Q* values of different copper ion concentrations (a), acid ion concentrations (b), chloride concentrations (c), the concentration of additives (d) and additive loss rules *Q* (e) and *Q*/*Q*_0_ (f).

Cyclic voltammetric stripping (CVS) was employed to optimize the fundamental electrolyte parameters for copper electrodeposition. The stripping charge (*Q*), which correlates with the deposition rate, was measured across varying concentrations of key components. As shown in Fig. 1a, *Q* increased with the Cu^2+^ concentration (20–90 g L^−1^), reaching the maximum of 15.8 mC at 85 g L^−1^. This concentration was identified as optimal, as higher levels promoted ionic competition. With Cu^2+^ fixed at 85 g L^−1^, the concentration of H_2_SO_4_ was varied from 70 to 100 g L^−1^. The *Q* value increased steadily, peaking at 28.41 mC with 90 g L^−1^ H_2_SO_4_ (Fig. 1b), indicating that this concentration optimally balanced enhanced conductivity against potential inhibitory effects. The influence of chloride ions was examined by adding 10–100 mg L^−1^ Cl^−^ to the optimized base electrolyte (85 g L^−1^ Cu^2+^ and 90 g L^−1^ H_2_SO_4_). The *Q* value decreased to the minimum of 25.52 mC at 20 mg L^−1^ Cl^−^ (Fig. 1c), demonstrating the strongest inhibition effect, which is crucial for deposit leveling. Consequently, 20 mg L^−1^ was selected as the optimal Cl^−^ concentration.

Furthermore, the individual effects of SPS, HEC, and collagen additives were evaluated in the optimized electrolyte. SPS functioned as an accelerator, increasing the *Q* value, with its effect saturating above 4 mg L^−1^. In contrast, both HEC and collagen acted as inhibitors, reducing the *Q* value. Collagen exhibited a significantly stronger suppression effect within a narrow active range of 0–6 mg L^−1^, while HEC showed a more gradual effect up to 14 mg L^−1^ (Fig. 1d). These distinct concentration-dependent behaviors provided the basis for formulating the subsequent ternary additive system.

In addition, the time-dependent consumption behaviors of the three additives (HEC, SPS, and collagen) during electrodeposition were quantitatively characterized *via* the evolution of the stripping charge (*Q*), as shown in Fig. 1e and f. Significant differences in the depletion kinetics and cumulative consumption were observed, reflecting their distinct functional mechanisms and adsorption dynamics. After 5 h of electrolysis, collagen exhibited the most rapid and pronounced consumption, with a Δ*Q* of 13.09 mC and a relative change (*Q*/*Q*_0_) of 52%. This indicates strong and likely irreversible adsorption or degradation at the electrode interface, consistent with its role as a potent but transient grain refiner. In contrast, SPS showed the largest absolute consumption (Δ*Q* = 3.79 mC, 64% change), yet its normalized *Q*/*Q*_0_ profile remained near 1.0, suggesting steady and predictable accelerator behavior. HEC demonstrated the slowest depletion (Δ*Q* = 1.42 mC, ∼10% change), reflecting the persistent adsorption typical of a polymeric suppressor. These divergent consumption patterns underscore the necessity of designing additive-specific replenishment strategies to maintain microstructural control and process stability in continuous copper foil production.

#### 3.1.2 Microstructure

**Fig. 2:**
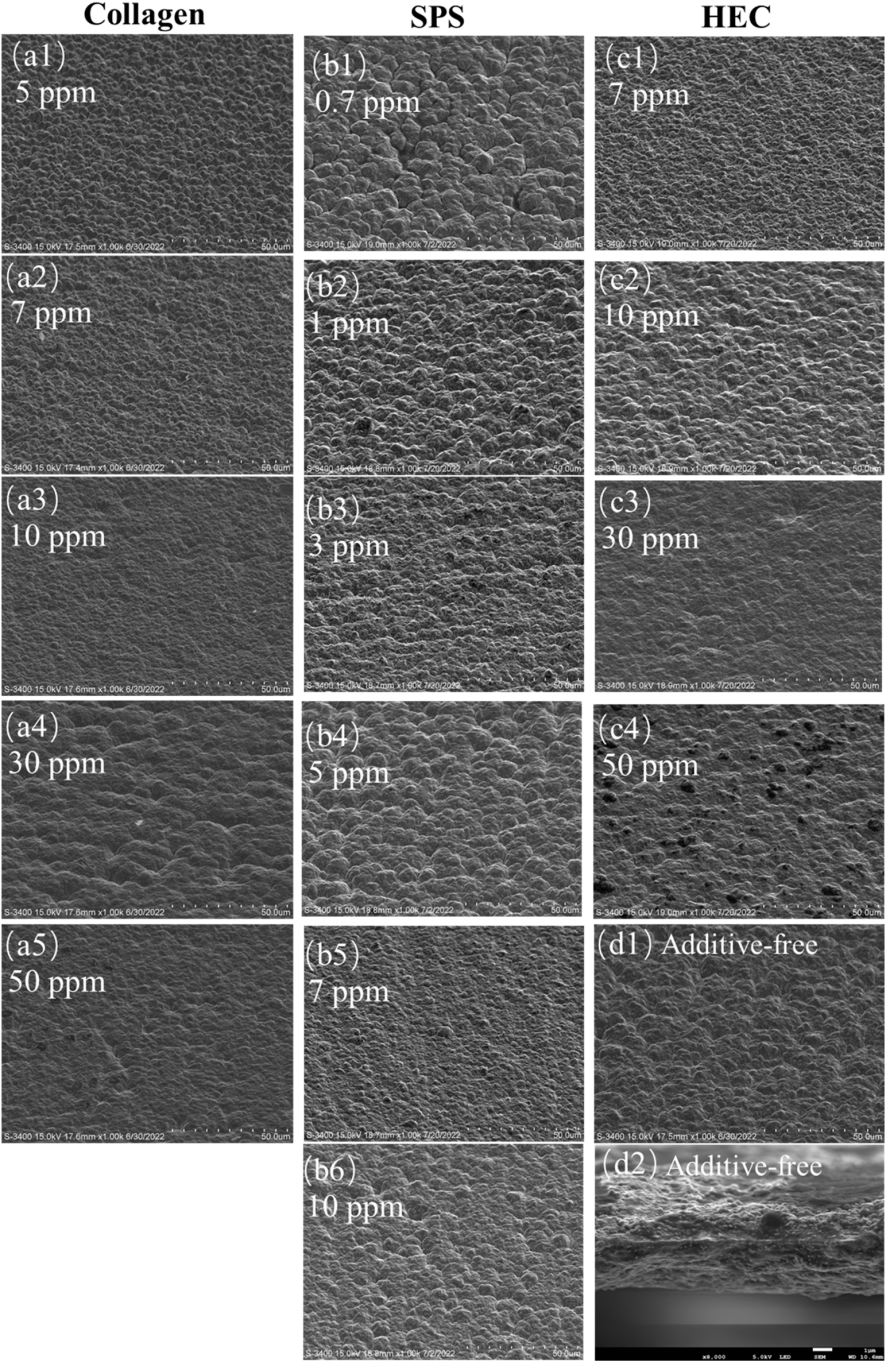
SEM morphology of the copper foils obtained by electrodeposition at different additives concentrations (a1–a5) collagen; (b1–b6) SPS; (c1–c4) HEC; and (d) additive free.

Fig. 2 quantitatively correlated the additive concentration gradients with distinct morphological transitions in the 6 µm ultrathin Cu foils. It was observed that the morphology of the copper foils was altered significantly by variations in the concentration of collagen. When no collagen was added to the system, the gross surface of the copper foils was composed of unevenly distributed clusters of small and large crystals (Fig. 2d), with concavities present between each cluster. The surface morphology comprising uneven crystal clusters (Fig. 2d1) was directly linked to the internal microstructure revealed in the cross-section image (Fig. 2d2). The foil exhibited a typical columnar growth pattern with low packing density and visible gaps between grains. This lack of consolidation and the prevalent structural defects inherent to additive-free deposition were identified as the root causes of its unsatisfactory mechanical strength and surface quality, highlighting the critical need for additive-induced microstructure control.

When collagen was introduced (Fig. 2a1–a5), a rapid reduction in grain size was achieved, and the copper foil surface was characterized by uniformly distributed dense, fine spiky grains. Upon increasing the concentration of collagen to 7 ppm, uneven refinement of the surface grains was observed with sporadic abnormally coarse grains, indicating that the additive could not be uniformly distributed across the cathode roll surface. With the further addition of collagen, a continuous reduction in grain size was observed until a concentration of 50 ppm was reached, where the surface grains became indistinguishable. A significant enhancement in surface flatness and grain refinement was demonstrated compared to the additive-free system. The grain refinement mechanism of collagen was attributed to its structural specificity. In acidic electrolyte, the cations were hydrolyzed by collagen. These cations migrated to the cathode plate surface and were adsorbed at the active sites, where the site resistance was increased. Consequently, the cathodic polarization during deposition was enhanced, leading to a reduced nucleation rate of copper grains. Simultaneously, localized grain growth was suppressed, preventing the formation of abnormally thick grains, thus achieving grain refinement.^17,27^

The addition of a small quantity of 0.7 ppm SPS resulted in coarse and loose copper grains, accompanied by visible inter-cluster pores. At this stage, SPS was not yet effective in grain refinement. When the concentration of SPS was increased to 1 ppm, grain refinement was observed, and the pores between clusters disappeared, indicating that SPS began to function at this concentration. To continue the experiment, the concentration of SPS was increased to 3 ppm. The copper foil surface exhibited a transition from conical to hill-shaped grains, with uniform deposition and no abnormally large particles. This hill-like transition was more pronounced at 0.005 g L^−1^ SPS. The change in morphology was attributed to the two-dimensional disc-shaped nucleation mode induced by the addition of SPS, where electrodeposited grains could only grow laterally along cusp grains or generate new surface nuclei. Lateral growth filled the depressions between spikes, resulting in inter-spike growth on the gross surface. Thus, to achieve a more uniform coating, the SPS dosage was increased to 7 ppm. A notable morphological change was observed, where the copper grain size was significantly reduced, the surface flatness was enhanced, and only a few coarse particles remained.

SPS and Cl^−^ were co-adsorbed on the electrode surface, increasing the Cl^−^ concentration near the cathode. This strengthened the “chlorine bridge effect”,^28,29^ facilitating more Cu^2+^ adsorption onto the cathode. Additionally, Cu^2+^ in the electrolyte formed surface complexes with SPS, which were adsorbed onto the copper particle peaks.^30,31^ Sulfonic acid groups captured Cu^2+^ and transferred them to particle valleys for reduction, filling the concave areas to create a smooth surface.^12^ This process accelerated the reduction of Cu^2+^ to Cu, increasing the Cu nucleation rate and density, thereby promoting grain refinement.

According to the gross surface morphology changes, an increase in fine, uniform copper particles was observed with an increase in the amount of HEC. Refinement was identified as a cumulative process, where without additives (Fig. 2d), spiky clusters of large particles dominated; at 7 ppm HEC, refinement was initiated with increased sharp-peaked small particles and uniform sizing, though large particles persisted. At 10 ppm HEC, the particles became rounded and smooth, but the grain size experienced a rebound. At 30 ppm HEC, the grain size further decreased, with no coarse particles and high flatness observed. However, at 50 ppm HEC, the surface appeared black due to partial oxidation and localized “over-suppression” caused by the excessive additive concentration.

**Fig. 3:**
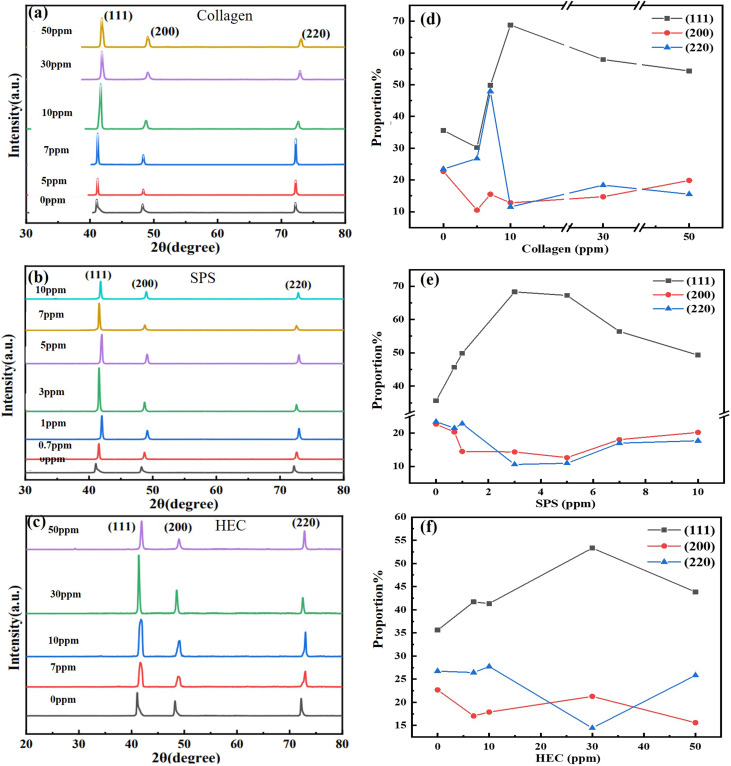
XRD patterns (a–c) and texture coefficients (d–f) of the copper foil obtained by electrodeposition at different collagen (a and d), SPS (b and e) and HEC concentrations (c and f).

X-ray diffraction analysis revealed that the crystallographic texture of the electrodeposited copper foils was highly sensitive to the specific additive chemistry and its concentration. The underlying mechanism involved competitive adsorption, which selectively altered the growth kinetics of different crystal planes. In the case of the collagen-modified foils (Fig. 3a), the diffraction intensities of the (111) and (220) planes exhibited a pronounced non-monotonic dependence on concentration. Quantitative texture coefficient (TC) analysis (Fig. 3d) showed that the (111) orientation dominated, with its TC peaking at 68.82% for the 10 ppm condition, which is nearly double the value of the additive-free foil (35.61%). This sharp texture evolution between 7 and 10 ppm collagen correlated directly with the steepest descent in the deposition rate observed by CVS (Fig. 1d), indicating that the rapidly intensified inhibition strength within this concentration window preferentially suppressed the growth of higher-energy planes such as (220), thereby thermodynamically favoring the development of the most stable (111) orientation. This signified the powerful, concentration-dependent ability of collagen to promote highly oriented growth, which was likely mediated by its specific adsorption on different surface sites, favoring the optimal coverage of the thermodynamically stable (111) plane.

Distinctly different behavior was observed with SPS (Fig. 3b). A sharp maximum in the (111) TC (68.30%) was induced by the accelerator SPS at a low concentration of 3 ppm, while the (200) and (220) orientations were concurrently suppressed (Fig. 3e). This was characteristic of a facet-selective acceleration mechanism. Beyond the optimum, the dominance of (111) waned, suggesting that excessive SPS led to a loss of growth selectivity, likely due to changes in the adsorption–desorption equilibrium or the onset of secondary inhibition effects.

In the case of the polymeric suppressor HEC (Fig. 3c), the texture evolution was more gradual. The TCs for the (111) and (200) planes reached a maximum at 30 ppm HEC, while the (220) TC was minimized (Fig. 3f). This trend aligned with the action of a leveling agent that imposed a general diffusion barrier, which disproportionately suppressed the growth of higher-energy planes such as (220), thereby relatively favoring the development of the (111) orientation. The inversion of this trend at higher HEC concentrations pointed to the saturation of adsorption sites or changes in the conformation of the polymer at the interface.

Collectively, these results demonstrated that each additive operated through a unique mechanism, *i.e.*, specific adsorption (collagen), accelerated reduction (SPS), and generalized suppression (HEC), to precisely control the crystallographic texture. This provided a fundamental basis for their synergistic use in the microstructure engineering of copper foils.

#### 3.1.3 Roughness

**Fig. 4:**
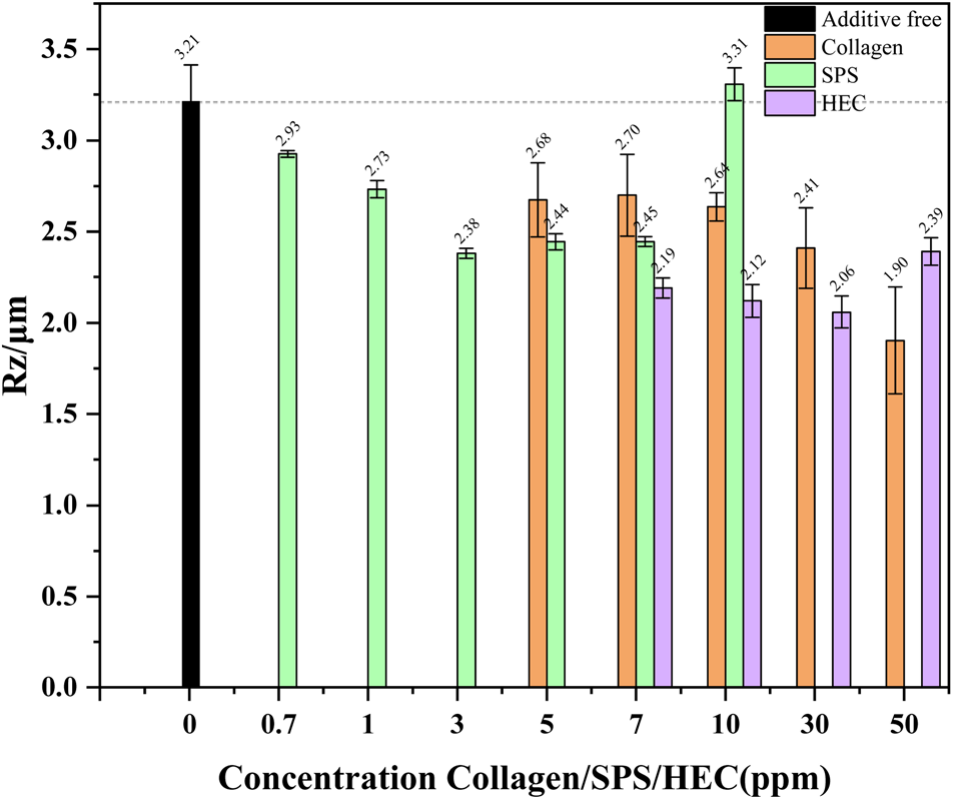
The surface roughness of copper foils obtained by electrodeposition at varying concentrations of collagen, SPS, and HEC.

The roughness variation curves of the copper foils prepared under different concentrations of collagen, SPS, and HEC were analyzed, as shown in Fig. 4. A pronounced reduction in roughness was observed, decreasing from approximately 3.3 µm for the additive-free foil to the minimum of 1.90 µm, 2.38 µm, and 2.06 µm for collagen, SPS, and HEC, respectively. This underscored their critical role in achieving microstructural uniformity. The incorporation of collagen induced a dramatic, monotonic decrease in roughness, reaching an optimal *R*_z_ of 1.90 µm at 50 ppm. In contrast, SPS exhibited a distinct zigzag variation, with an optimal value of 2.38 µm at 3 ppm. This non-monotonic behavior was characteristic of an accelerator whose effectiveness was concentration-dependent. At the optimal concentration, SPS promoted sufficient bottom-up filling in valleys to level the surface. However, deviations from this concentration upset the balance between acceleration and inhibition, leading to a resurgence of uneven growth and increased roughness, as reflected in the morphological transition from sharp conical to hill-shaped grains. The roughness profile for HEC displayed a stepwise decreasing trend, eventually reaching a minimum of 2.06 µm at 30 ppm. This pattern indicated a polymeric suppressor governed by cumulative adsorption. Each incremental increase in HEC concentration allowed more polymer chains to adsorb onto the cathode surface, enhancing the diffusion barrier and improving the leveling. The distinct roughness–concentration relationships of monotonic (collagen), volcano-type (SPS), and stepwise (HEC) directly mirrored their fundamental operating mechanisms, *i.e.*, site-specific inhibition, controlled acceleration, and cumulative suppression, respectively. This clear mechanistic link between adsorption behavior and macroscopic topography is crucial for designing synergistic additive formulations.

#### 3.1.4 Mechanical properties

**Fig. 5:**
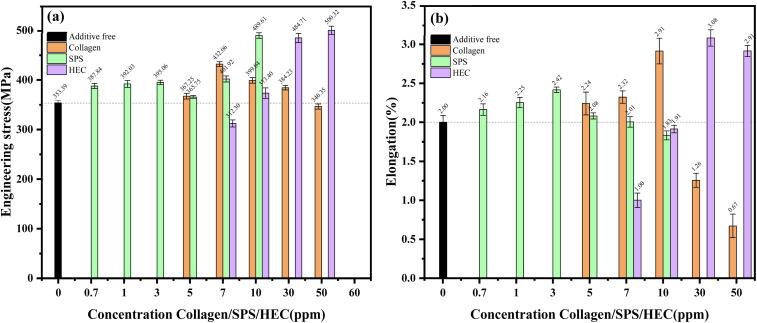
(a) The elongation of copper foils obtained by electrodeposition at varying concentrations of collagen, SPS, and HEC. (b) The tensile strength of copper foils obtained by electrodeposition at varying concentrations of collagen, SPS, and HEC.

The mechanical properties of the electrodeposited copper foils, particularly tensile strength and elongation, were critically influenced by the specific additive-induced microstructural evolution, as quantified in Fig. 5. The data revealed distinct structure–property relationships governed by the unique mechanism of each additive. The collagen-modified foils exhibited a pronounced strengthening effect, with their tensile strength increasing from a baseline of ∼353 MPa (additive-free) to a peak of approximately 430 MPa at 7 ppm. This enhancement was attributed to the formation of refined, conical grains and enhanced intergranular bonding, coinciding with a maximum in the (220) texture coefficient. The concurrent decline in both (220) orientation and tensile strength at 10 ppm collagen suggested a direct correlation between (220)-oriented growth and strength improvement, which is likely due to its higher resistance to dislocation slip. In the case of SPS, the tensile strength displayed a dual-peak behavior, *i.e.*, an initial peak of ∼395 MPa at 3 ppm, followed by a reduction at 5 ppm, associated with coarse, hill-shaped grains. A second peak of ∼490 MPa emerged at 10 ppm, where extreme grain refinement promoted strength but surface pits compromised the ductility, highlighting a strength-ductility trade-off. In contrast, the addition of HEC facilitated a monotonic strengthening trend, reaching a maximum of ∼500 MPa at 50 ppm, aligned with progressive grain refinement and surface smoothness.

Analysis of elongation (Fig. 5b) further elucidated the deformation behavior. Collagen improved the ductility to the maximum at 7 ppm, but embrittlement occurred at 30 ppm, where coarse grain clusters acted as stress concentration sites. In the case of SPS, peak elongation coincided with a uniform fine-grained structure and strong (111) texture at 3 ppm. Notably, HEC achieved the most substantial ductility enhancement (∼3.1% at 30 ppm), combining minimal grain size, low roughness, and dominant (111) orientation. These distinct mechanical responses underscored the specific actions of each additive, where collagen provided strengthening through texture control, SPS imposed a concentration-dependent trade-off, and HEC enabled the simultaneous improvement of both properties. These findings established a framework for understanding the synergistic potential in ternary additive systems.

### 3.2 Microstructure and performance regulation of copper foils *via* ternary additives

#### 3.2.1 Surface morphology

**Fig. 6:**
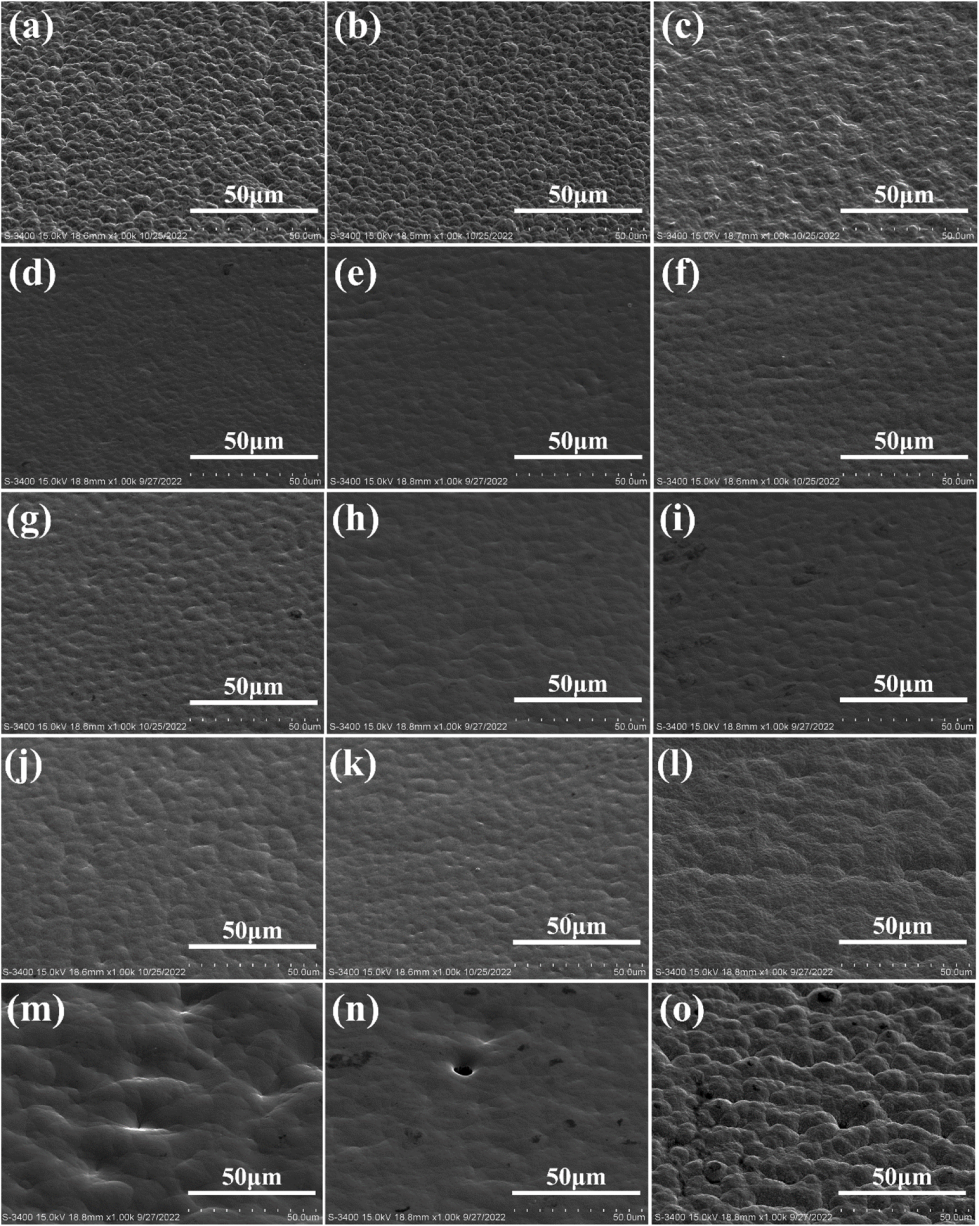
SEM morphology of copper foil obtained using the SPS–HEC–collagen ternary composite. (a) T1; (b) T2; (c) T3; (d) T4; (e) T5; (f) T6; (g) T7; (h) T8; (i) T9; (j) T10; (k) T11; (l) T12; (m) T13; (n) T14; and (o) T15.

The surface morphology of copper foils from sixteen composite additive experiments (T0–T15) was characterized, as shown in Fig. 6. Significant morphological improvements were observed following the integration of the ternary additive system. In formulations T1–T6, where the SPS concentration was fixed at 0.7 ppm, the grain refinement and enhanced surface flatness were correlated with increasing concentrations of collagen and HEC. In contrast, severe morphological deterioration, characterized by depressions, pinholes (T13–T14), and loose globular clusters (T15), was observed at elevated additive levels.

**Fig. 7:**
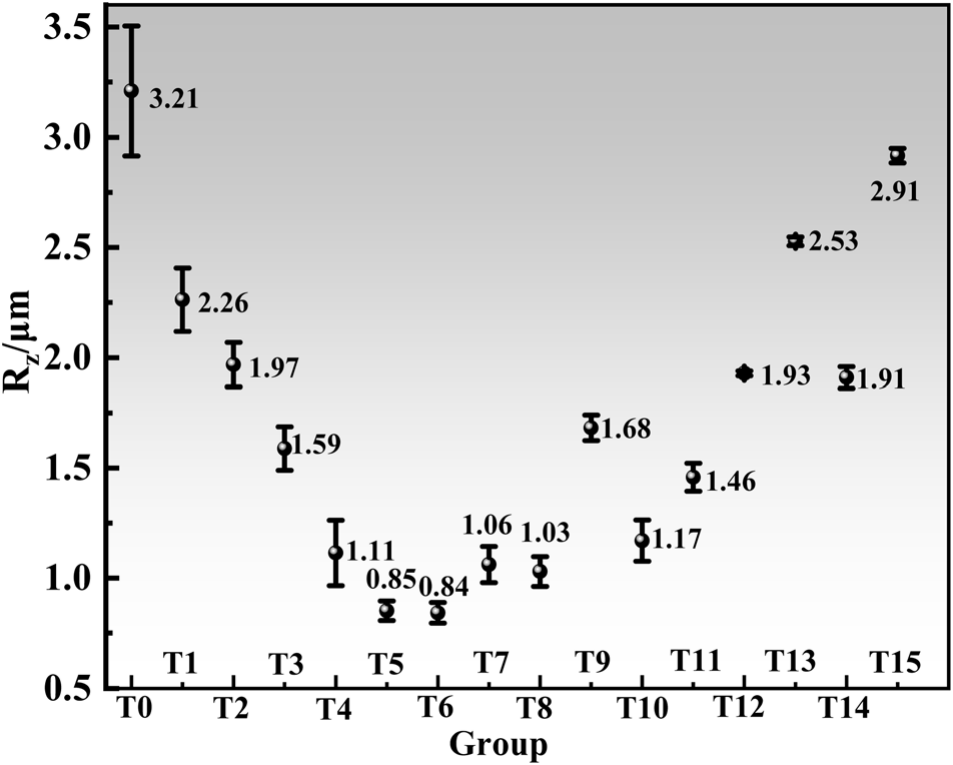
Surface roughness of the copper foil with SPS–HEC–collagen ternary composite.

The surface roughness of the T0–T15 series was further elucidated in Fig. 7. A continuous decrease in roughness was recorded from T0 to T6, reaching a minimum arithmetic mean roughness (*R*_z_) of 0.84 µm at T6. The subsequent increase in roughness was linked to higher concentrations of SPS, collagen, and HEC, culminating in a peak *R*_z_ of 2.91 µm at T15. These results demonstrated that the additives enabled precise regulation of copper ion deposition on the cathode surface.

#### 3.2.2 XRD analysis

**Fig. 8:**
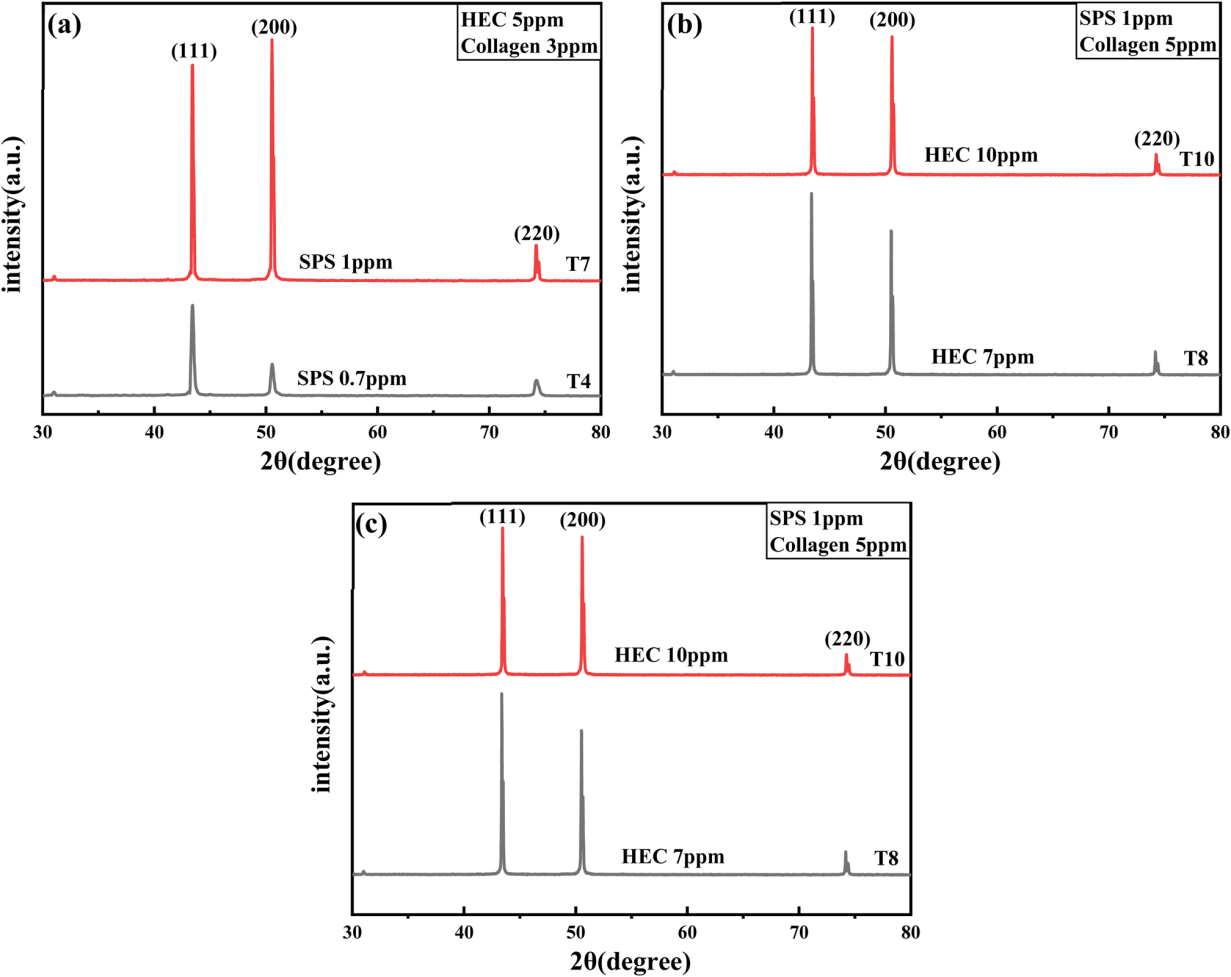
XRD patterns of copper foil obtained using SPS–HEC–CO ternary composite. (a) Comparison of T4 and T7; (b) comparison of T9 and T10; and (c) comparison of T8 and T10.

**Fig. 9:**
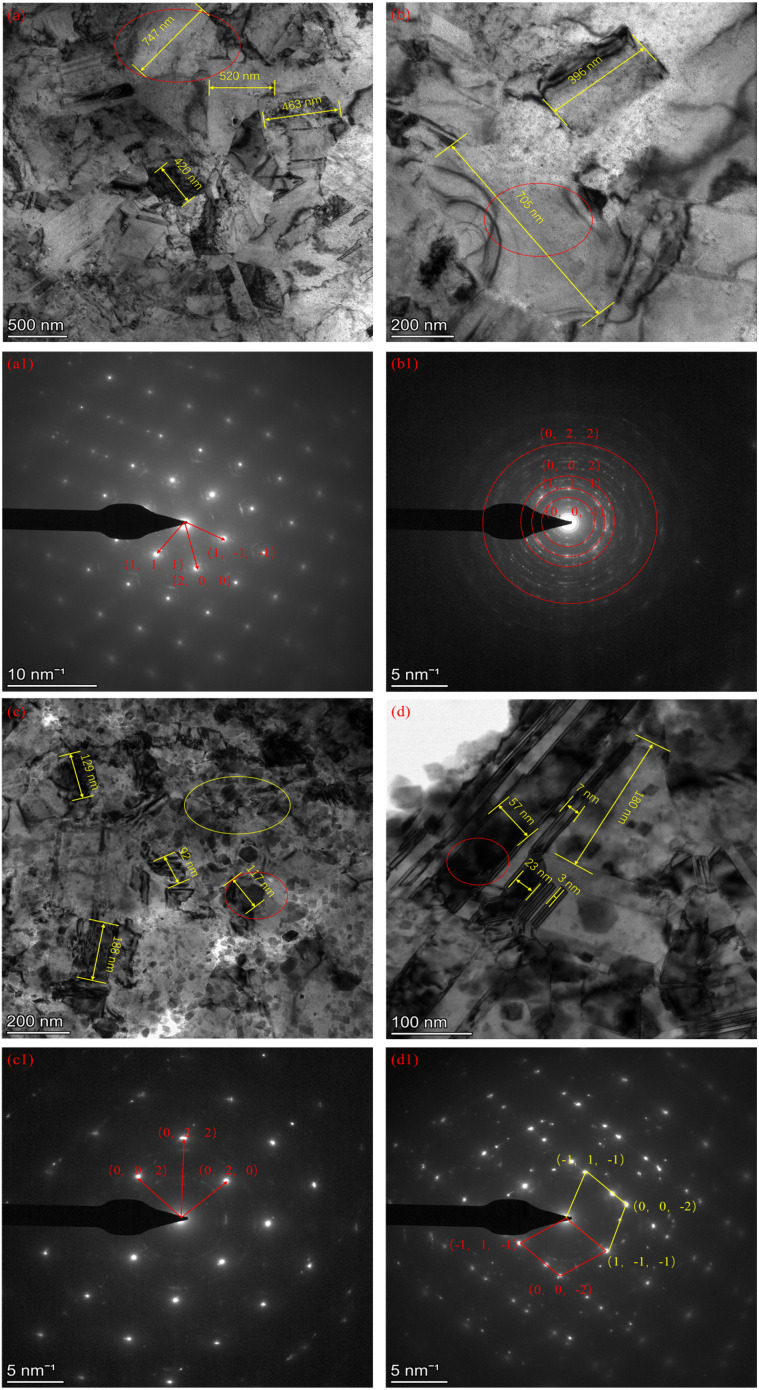
TEM of copper foils in the presence of T0 additive-free and T4 composite additives. (a and b) T0 additive-free; (a1 and b1) diffraction patterns of T0 additive free copper foils; (c and d) T4 composite additives; and (c1 and d1) diffraction patterns of T4 composite additive copper foils.

X-ray diffraction analysis was performed on copper foils electrodeposited from electrolytes containing different composite additive formulations. Four sample sets were characterized, and their diffraction patterns are presented in Fig. 8. The influence of increasing concentrations of SPS, collagen, and HEC on the crystalline orientations was systematically evaluated, as shown in Fig. 8a–c. In the ternary additive system with HEC fixed at 5 ppm and collagen at 3 ppm (Fig. 9a), a dramatic increase in diffraction peak intensity for the (111) and (200) crystal planes was observed when the SPS concentration was increased to 1 ppm. The (200) plane was established as the preferential orientation under these conditions. As shown in Fig. 8b, with SPS maintained at 1 ppm and HEC at 5 ppm, the diffraction peak intensity of the (200) plane was reduced relative to the (111) plane as the collagen content was elevated. Further investigation (Fig. 8c) demonstrated that preferential orientation could be altered by varying the HEC concentration when SPS was fixed at 1 ppm and collagen at 5 ppm, where at 7 ppm HEC, the (111) plane was distinctly preferred, while at 10 ppm HEC, comparable diffraction peak intensities were recorded between the (111) and (200) planes.

#### 3.2.3 TEM analysis

The microstructure of the copper foils was examined using transmission electron microscopy (TEM). Comparative analysis was conducted between foils deposited with the T4 composite additive formulation and additive-free reference samples (T0), as illustrated in Fig. 9. Significant microstructural modifications were observed following the incorporation of the additives. Pronounced grain refinement was achieved, indicating a substantial reduction in grain size mediated by the composite additive system. The diffraction patterns of the additive-free copper foils were identified as the characteristic (111) and (200) lattice planes of face-centered cubic (FCC) copper (Fig. 9a1), consistent with the XRD data presented in Fig. 3 and 8. As shown in Fig. 9c and d, distinct twinning structures were identified through analysis of the red-circled areas. The diffraction pattern was classified as comprising both single-crystal and twin components, with a twin/matrix layered structure observed in the majority of grains due to the high-density twin boundaries. Copper, as an FCC metal with low intrinsic stacking fault energy, readily forms growth twins. The additives were demonstrated to refine the crystal structure, significantly increasing the twin boundary density and forming staggered high-density dislocation zones. This microstructural evolution hindered plastic deformation, while improving the tensile strength. Consequently, a direct correlation was established between the additive-induced microstructural changes and enhanced mechanical properties. The composite additives facilitated simultaneous grain size reduction, alteration of preferential grain orientation, and promotion of twin boundary formation, collectively contributing to the superior mechanical performance of the copper foils.

#### 3.2.4 Mechanical properties

**Fig. 10:**
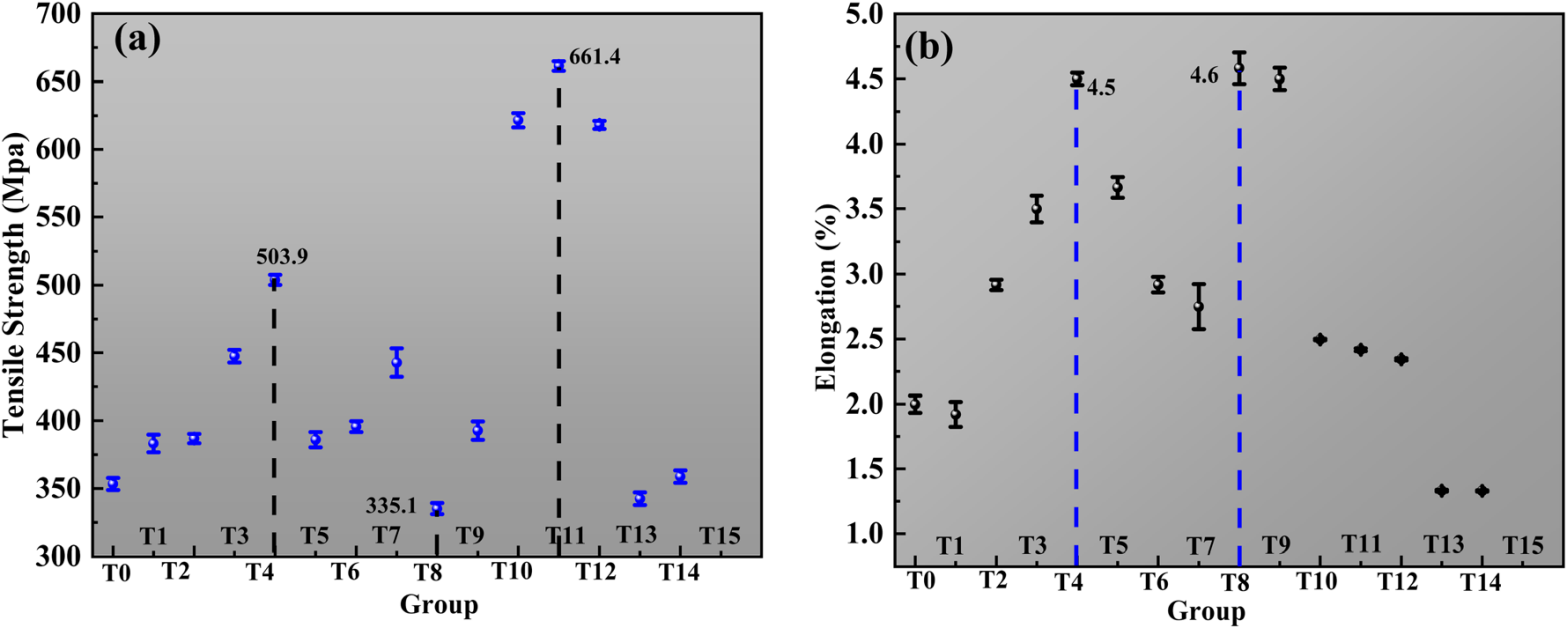
Tensile strength (a) and elongation (b) of copper foil in the SPS–HEC–CO ternary composite.

The mechanical properties of the copper foils electrodeposited with the ternary additive system exhibited significant dependence on the compositional parameters, as summarized in Fig. 10. The tensile strength response (Fig. 10a) complemented these findings, revealing distinct optimization pathways. In the 0.7 ppm SPS series, the strength reached the maximum of 503.85 MPa at T4, corresponding to the peak ductility point, demonstrating the possibility of synergistic enhancement. In contrast, the 1 ppm SPS series achieved a higher peak strength of 661.36 MPa at T11, but this was coupled with significantly reduced elongation, highlighting the classic strength-ductility trade-off. The 3 ppm SPS series resulted in lower overall strength values, confirming that an excessive accelerator concentration disrupts the balanced microstructure required for optimal mechanical performance.

Analysis of elongation (Fig. 10b) revealed a non-monotonic response to additive concentration. When the SPS concentration was maintained at 0.7 ppm, a simultaneous increase in collagen and HEC from T1 to T4 progressively enhanced the ductility, reaching the maximum elongation of 4.50% in the T4 formulation (3 ppm collagen and 5 ppm HEC). This optimal combination promoted a microstructure conducive to homogeneous plastic deformation. A further increase in the concentration of collagen and HEC beyond this point (T5 and T6) led to a pronounced decrease in elongation, indicating the onset of embrittlement due to additive oversaturation. A more complex behavior emerged with increasing SPS concentrations. At 1 ppm SPS (T7–T12), elongation again displayed an initial increase, peaking at 4.58% for T8 (5 ppm collagen, 7 ppm HEC), followed by a subsequent decline. Notably, when the SPS concentration was elevated to 3 ppm (T13–T15), the elongation was substantially suppressed across all collagen/HEC combinations, achieving a minimum of only 1.24%, which underscores the dominant role of SPS in accelerating the deposition kinetics, often at the expense of ductility. Cross-correlation of the mechanical data with microstructural analysis provided mechanistic insights. The superior combination of strength (503.85 MPa) and elongation (4.50%) achieved by the T4 formulation was attributed to its refined, twinned grain structure (Fig. 9c and d). This microstructure enhances the strength *via* the Hall–Petch mechanism and twin-boundary strengthening, while facilitating uniform deformation. The T11 formulation, which prioritized strength, likely developed an extremely refined but less ductile microstructure. The poor performance of the high SPS concentration samples (*e.g.*, T15) aligned with a disordered, potentially defective microstructure. Therefore, the T4 composition (0.7 ppm SPS, 3 ppm collagen, and 5 ppm HEC) was identified as the optimal formulation, achieving an enhanced balance of mechanical properties through microstructural control. To further contextualize the mechanical performance, a direct comparison was made with commercially available copper foils.^32^ The commercial foils exhibited a considerable spread in properties, with tensile strength ranging from 318.05 MPa to 508.73 MPa and elongation varying from 2.95% to 7.45%. This variability underscores the challenge in consistently achieving a superior balance of strength and ductility in industrial production. In contrast, our optimized T4 foil (0.7 ppm SPS, 3 ppm collagen, and 5 ppm HEC) delivered a synergistic combination of high tensile strength (503.85 MPa) and exceptional elongation (4.50%), effectively bridging the performance gap observed in commercial benchmarks. Notably, our T11 formulation (1 ppm SPS, 7 ppm collagen, and 30 ppm HEC) achieved a remarkable tensile strength of 661.36 MPa, significantly surpassing all commercial samples, albeit with a trade-off in ductility. The superior and predictable performance of our foils was fundamentally attributed to the microstructural control enabled by the synergistic additive interactions, as detailed in the analysis in Fig. 10. Unlike conventional foils, which likely possess more heterogeneous microstructures, our approach consistently produced a refined grain morphology with a high density of twin boundaries, as confirmed by TEM (Fig. 9c and d). This controlled microstructure was the key to achieving enhanced mechanical properties that meet or exceed current industry standards, highlighting the practical significance and potential impact of our methodology for advanced battery current collectors.

#### 3.2.5 Synergistic additive mechanisms and industrial outlook

During the electrodeposition process, the copper foil surface typically exhibited non-planar characteristics, a phenomenon attributed to the “peak discharge” effect that created potential differences between peak and valley regions. The peak current density measurements consistently exceeded the valley current density values, resulting in preferential metal ion reduction at the surface protrusions. This preferential deposition mechanism led to accelerated metal growth at elevated features, ultimately contributing to increased surface roughness and morphological heterogeneity. Through systematic investigation, the additives were classified into two primary functional categories based on their electrochemical behavior, *i.e.*, promoters and inhibitors. The accelerator SPS (3,3′-dithiodipropanesulfonic acid disodium salt) promoted the formation of smooth, planar copper layers through sulfhydryl group adsorption and Cu^2+^ transfer mechanisms. Notably, the SPS–Cu^+^ complexes functioned as effective barriers that constrained ion deposition channels, accelerating grain growth within depressions relative to protrusions. Chloride ions (Cl^−^) primarily facilitated adsorption at high-energy cathodic interfaces, inducing modifications in electrode structure and bilayer configuration. The collagen additive, existing as ammonium cations in acidic solutions, demonstrated exceptional grain refinement capabilities through electrostatic attraction to the negatively charged surface protrusions. The formed adsorption layer effectively hindered subsequent copper ion nucleation, while the electrostatic interactions between the positively charged nitrogen of collagen and sulfonic acid groups of SPS revealed synergistic potential. HEC (hydroxyethyl cellulose) exhibited superior surface leveling through cumulative adsorption effects, with each incremental concentration increase enhancing the diffusion barrier and promoting sudden improvements in surface flatness. The ternary additive system demonstrated remarkable synergistic effects, achieving a 229.0% improvement in elongation (to 4.58%), 188.2% enhancement in tensile strength (to 661.4 MPa), and 73.8% reduction in surface roughness (*R*_z_ to 0.84 µm) compared to additive-free foils. The microstructural analysis revealed that SPS promoted densification and morphological transformation from conical to hill-like grains, collagen enabled ultra-grain refinement, while HEC dominated mechanical enhancement through superior refinement and flattening mechanisms (Table 2).

**Table 2:** Effects of different additive compounds on electrolytic properties of copper foil

Groups	SPS (ppm)	Collagen (ppm)	HEC (ppm)	Tensile strength (MPa)	Elongation (%)	Knitting factor			Roughness
						(111)	(200)	(220)	
T4	0.7	3	5	503.86	4.5	65.2	22.2	12.5	1.11
T7	1			442.74	2.75	42.5	50.1	7.5	1.06
T10	1	5	10	621.52	2.5	44.7	47.6	7.7	1.17
T9		7		392.8	4.5	55.5	36.8	7.7	1.68
T8	1	5	7	335.14	4.58	47.9	44.7	7.5	1.03
T10			10	621.52	2.5	44.7	47.6	7.7	1.17

This research establishes a fundamental framework for understanding the additive synergy in copper electrodeposition, highlighting the critical importance of microstructure control in advancing battery technology. The growing demand for high-energy-density lithium-ion batteries necessitates the development of ultrathin copper foils with enhanced mechanical properties, positioning additive engineering as a pivotal strategy for next-generation current collector design. Future research directions should focus on optimizing additive replenishment strategies for continuous production and exploring advanced characterization techniques to further elucidate the nanoscale interaction mechanisms. Furthermore, future work will include long-term cycling performance in full-cell configurations to fully assess practical durability.

## 4. Conclusion

Herein, the effects of SPS, collagen and HEC additives on the microstructure and properties of electrolytic copper foils were investigated, and the role of additives in the electrodeposition process was discussed.

In this study, the synergistic effects of ternary additives (SPS, collagen, and HEC) on the microstructure and mechanical properties of electrolytic copper foils were systematically investigated, and the underlying mechanisms governing the additive interactions in the electrodeposition process were elucidated.

(1) The significant synergistic effects of ternary additives (SPS, collagen, and HEC) were demonstrated, resulting in high-performance electrolytic copper foils with a tensile strength of 661.4 MPa, elongation of 4.58%, and roughness of 0.84 µm, surpassing single-component additive systems in comprehensive properties.

(2) Microstructural transformation was induced by additives, where SPS promoted densification of the copper foil morphology from loose conical to compact hilly structures; collagen and HEC were identified as effective grain refiners, enhancing the surface flatness and preferentially promoting (111) crystal plane growth.

(3) Mechanical property enhancement was achieved through additive-mediated grain control, where the grain occupancy of the (111) and (220) planes increased, while growth along the (200) plane was suppressed, thereby optimizing the tensile strength–elongation balance.

(4) Twin grain boundary formation was facilitated by additives, attributed to the low stacking fault energy of FCC copper and additive-induced inhibition of dislocation motion, which contributed to simultaneous strength–toughness improvement.

(5) This study provides a scalable, cost-effective strategy for the industrial production of high-performance 6 µm Cu foils.

## Author contributions

Jinze Zhang: writing – original draft; project management. Jialin Li: writing – original draft; project management. Liping Wang: writing – review and editing. Zhongbo Bai: methodology. Eryong Liu: supervision; funding acquisition; writing – review and editing. Hui Cai: data curation. Jingli Zhang: funding acquisition; investigation. Qinhao Yang: software.

## Conflicts of interest

The authors declare that they have no known competing financial interests or personal relationships that could have appeared to influence the work reported in this paper.

## Data Availability

The data that support the findings of this study are available from the corresponding author upon reasonable request.

## References

[cit1] Yi G., Cai F., Peng W., He T., Yang X., Huang Y., Yuan Z., Wang P. (2012). Eng. Failure Anal..

[cit2] Quinet M., Lallemand F., Ricq L., Hihn J.-Y., Delobelle P., Arnould C., Mekhalif Z. (2009). Electrochim. Acta.

[cit3] Cheng X. , Li Y. F., Huang G. J., Yin X. Q., Li Y. Z., Yao E. D., Ma X. L., Xie X. S., Qi S. L. and Li Z. M., Materials Science Forum, 2019, vol. 944, pp. 205–211

[cit4] Wang R., Liu Z., Yan C., Qie L., Huang Y. (2023). Acta Phys.-Chim. Sin..

[cit5] Zhou B., Bonakdarpour A., Stoševski I., Fang B., Wilkinson D. P. (2022). Prog. Mater. Sci..

[cit6] Xiao Z. e., Chen J., Liu J., Liang T., Xu Y., Zhu C., Zhong S. (2019). J. Power Sources.

[cit7] Li S., Zhu Q., Zheng B., Yuan J., Wang X. (2019). Mater. Sci. Eng., A.

[cit8] Liu Z., Huang W., Xiao Y., Zhang J., Kong W., Wu P., Zhao C., Chen A., Zhang Q. (2024). Acta Phys.-Chim. Sin..

[cit9] Emekli U., West A. C. (2010). J. Electrochem. Soc..

[cit10] Hai N. T. M., Broekmann P. (2015). ChemElectroChem.

[cit11] Yang L., Weng W., Zhu H., Chi X., Tan W., Wang Z., Zhong S. (2023). Mater. Today Commun..

[cit12] Liao J., Wang L., Song N., Huang J., Zhao M., Zhao M., Tang Y., Tan Y., Fan X. (2024). Mater. Sci. Eng., B.

[cit13] Zhang R., Yang S., Qin S., Wang P., Wang W., Mitsuzaki N., Chen Z. (2023). Cryst. Res. Technol..

[cit14] Ariannezhad M., Habibi D., Heydari S. (2019). Polyhedron.

[cit15] Chan P.-F., Ren R.-H., Wen S.-I., Chang H.-C., Dow W.-P. (2017). J. Electrochem. Soc..

[cit16] Turner D., Johnson G. (1962). J. Electrochem. Soc..

[cit17] Chang T., Jin Y., Wen L., Zhang C., Leygraf C., Wallinder I. O., Zhang J. (2016). Electrochim. Acta.

[cit18] Moffat T. P., Wheeler D., Josell D. (2004). J. Electrochem. Soc..

[cit19] Vereecken P. M., Binstead R. A., Deligianni H., Andricacos P. C. (2005). IBM J. Res. Dev..

[cit20] Nagayama T. , Yoshida H. and Shohji I., International Electronic Packaging Technical Conference and Exhibition, American Society of Mechanical Engineers, 2013, vol. 55751, p. V001T07A012

[cit21] Wang S. P., Wei K. X., Wei W., Du Q. B., Alexandrov I. V. (2022). Phys. Status Solidi A.

[cit22] Sun Y., Pan J., Liu L., Fang Y., Han G., Liu J. (2022). J. Appl. Electrochem..

[cit23] Woo T.-G., Park I.-S., Seol K.-W. (2013). Electron. Mater. Lett..

[cit24] Woo T.-G., Park I.-S., Seol K.-W. (2013). Electron. Mater. Lett..

[cit25] Yu W., Lin C., Li Q., Zhang J., Yang P., An M. (2021). J. Appl. Electrochem..

[cit26] Zhang H., Gu Z.-Y., Wang X.-T., Zhao X.-X., Heng Y.-L., Liu Y., Yang J.-L., Zheng S.-H., Wu X.-L. (2024). Adv. Mater..

[cit27] Meudre C., Ricq L., Hihn J.-Y., Moutarlier V., Monnin A., Heintz O. (2014). Surf. Coat. Technol..

[cit28] Ren P., An M., Yang P., Zhang J. (2022). Appl. Surf. Sci..

[cit29] Gu M., Li Q., Fu B.-H., Xian X.-H. (2010). Trans. IMF.

[cit30] Dow W.-P., Huang H.-S. (2005). J. Electrochem. Soc..

[cit31] Tan M., Guymon C., Wheeler D. R., Harb J. N. (2006). J. Electrochem. Soc..

[cit32] Yang J., Wang L., Bai Z., Peng X., Feng B., Liu E. (2024). Met. Mater..

